# Fc gamma receptor polymorphisms in systemic lupus erythematosus and their correlation with the clinical severity of the disease

**DOI:** 10.4103/0971-6866.44998

**Published:** 2008

**Authors:** Vandana Pradhan, Manisha Patwardhan, K. Ghosh

**Affiliations:** Department of Immunobiology, National Institute of Immunohaematology, 13^th^ Floor, KEM Hospital Building, Parel, Mumbai 400 012, India

**Keywords:** Systemic Lupus Erythematosus (SLE), Fc gamma receptors (Fc γ Rs), IgG, Fc γ RIIA, Fc γ R IIB, Fc γ R IIIA, Fc γ R IIIB

## Abstract

Receptors for the Fc domains of IgG (Fc γ R) play a critical role in linking humoral and cellular immune responses. The various Fc γ R genes may contribute to differences in infectious and immune related diseases in various ethnic populations. Polymorphisms of Fc γ R mainly Fc γ R IIA, IIB, IIIA, IIIB have been identified as genetic factors influencing susceptibility to disease or disease course of a prototype autoimmune disease like Systemic Lupus Erythematosus (SLE). Activated and inhibitory Fc γ Rs seem to play an important role in the pathogenesis of SLE, in initiation of autoimmunity, the subsequent development of inflammatory lesions and finally immune clearance mechanisms. This review focuses on the role of Fc γ R polymorphism and their association with clinical manifestations and initiation of autoantibody production, inflammatory handling of immune complexes and disease development in SLE patients.

Many cells feature membrane glycoproteins called Fc receptors (FcR) that have an affinity for the Fc portions of secreted antibody molecules. These receptors are responsible for the movement of antibodies across cell membranes and transfer of IgG from the mother to the fetus across the placenta. These receptors allow passive acquisition of antibodies by many cell types, including B and T lymphocytes, neutrophils, mast cells, eosinophils, macrophages, and natural killer cells. Engagement of antibody-bound antigens by the Fc receptors of macrophages or neutrophils provides an effective signal for the efficient phagocytosis of Ag-Ab complexes.

There are many different Fc receptors. The poly-Ig receptor is essential for the transport of polymeric immunoglobulins (polymeric IgA and pentameric IgM) across epithelial surfaces. In humans, the neonatal Fc receptor (FcRn) transfers IgGs from the mother to the fetus during gestation and also plays an important role in the regulation of IgG serum levels. Fc receptors have been discovered for many of the Ig classes. There is an Fc γ receptor that binds to IgA, an Fc γ receptor that binds to IgE, and several varieties of Fc γ receptors (RI, RII-A, RII-B1, RII-B2, RIII) capable of binding IgG and its subclasses. The cross-linking of Fc receptors by binding of Ag-Ab complex results in the initiation of signal transduction cascades that results in phagocytosis or antibody-dependent cell mediated cytotoxicity (ADCC).[1]

Systemic lupus erythematosus (SLE) is a polygenic disorder of de-regulated inflammation. Numerous specific candidate genes have been identified and most relate to the handling of immune complexes or Ag presentation. The Fc receptor (Fc R IIA and Fc R IIIA) gene in particular and genetic variations in these receptors mainly affect these functions adversely. This results in an amplified loss of both B and T cell tolerance. Fc receptors thus play a critical role in linking the cellular and humoral immunity. Various Fc γ receptor genotypes are suspected to contribute to differences in immune mediated diseases like SLE in various ethnic populations.[2,3]

Fc γ receptors are critically involved at multiple stages of an immune response ranging from antigen presentation and regulation of antibody production to the end stage effector mechanisms of inflammation. IgG autoantibodies that are detectable in the majority of autoimmune diseases are ligands for Fc γ receptors. Fc receptors constitute large family genes belonging to the family of multi-chain immune recognition receptors. The three classes Fc γ RI, Fc γ R II, and Fc RIII vary in their antibody affinity, cellular expression, and in vivo function. Fc γ receptors are critically involved at multiple stages of an immune response, ranging from antigen presentation and regulation of antibody production to the end stage effector mechanisms of inflammation [Figure 1].[4–6]

**Figure 1:**
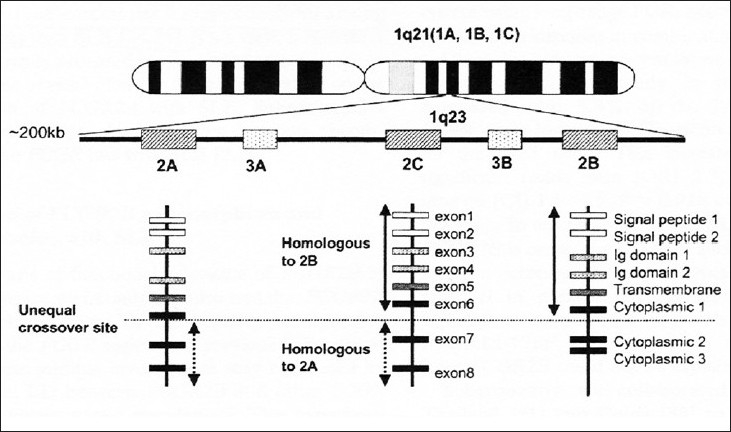
Human immunoglobulin G receptors

Fc γ RI (CD64), a high affinity receptor expressed on monocytes, macrophages, neutrophils, and dendritic cells, is comprised of isoforms IA and IB. Fc γ RI has a high affinity for monomeric human IgG1 and IgG3. Fc γ RI does not show genetic polymorphism. Fc γ RI, Fc γ RIIA, and Fc γ RIIIA are activating receptors, characterized by the presence of an immuno receptor tyrosine-based activation motif (ITAM). In contrast, Fc γ RIIIB is a GPI-linked receptor found only in humans and is thought to be a neutrophil specific decoy receptor able to bind IgG immune complexes without triggering activation. In the case of Fc γ RIIA, a receptor unique to humans, the ITAM-motif is present in the cytoplasmic tail of the receptor. The diverse functions of these activating receptors together regulate a large portion of antibody-dependant inflammatory processes and therefore will probably have an important role in autoantibody mediated damage in SLE.[7]

Fc γ RII (CD32) is a low affinity receptor present on phagocytic cells, B cells, and dendritic cells. Fc γ RII is comprised of isoforms IIA, IIB1, IIB2, IIB3, and IIC. Fc γ RIIA has two codominantly expressed alleles R131 and H131 and they exhibit significant population polymorphism, the 131-Arg (R131) allele binding IgG2 much less avidity than the 131-His (H131) allele. R/R 131 homozygosity has been associated with severe disease manifestations, renal involvement, and an early onset of disease in SLE patients. Since immune complex clearance is essential in SLE, Fc γ. RIIA genes are thought to be important disease susceptibility factors for SLE, particularly lupus nephritis.[4,6] It remains to be determined whether Fc γ RIIA polymorphism may play a critical role in certain groups of patients. Polymorphisms in Fc γ RIIA may also be important in determining disease phenotype and identification of this influence may have important implications in patient care and in identifying patients for more aggressive therapy.[8]

Fc γ RIIB is an inhibitory receptor carrying an immuno receptor tyrosine-based inhibition motif (ITIM) in its cytoplasmic domain. Its isoforms Fc γ RIIB1 and Fc γ RIIB2 are encoded by the same gene, but show distinct cell type distribution and functions. Fc γ RIIB1 is exclusively expressed on B cells and, upon cross-linking with the B-cell antigen receptor (BCR) by IgG containing immune complexes (ICs), it acts as negative feedback regulator by inhibiting BCR-elicited activations signals. Alternative splicing of the first intra-cytoplasmic exon results in Fc γ RIB2, which is mainly expressed on macrophages, where it internalizes bout IgG-containing ICs enabling antigen presentation [Figure 2].[9]

**Figure 2:**
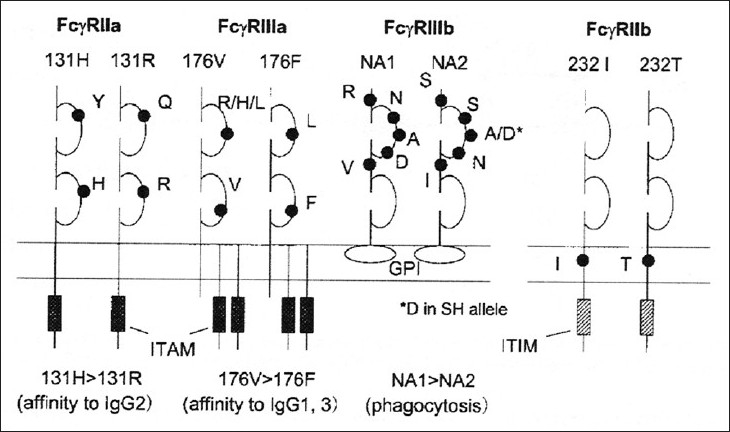
The structure of human Fc γ receptors : Fc γ R I, Fc γ RII and Fc γ RIII (Picture taken from Salmon and Pricop,2001)

Fc γ RIII (CD16) encodes a single polymorphic protein, which is expressed on Natural Killer (NK) cells and monocytes. It has two isoforms IIIA and IIB. The wild-type sequence at position 176 encodes a phenylalanine (176-F), whereas the polymorphic variant is 176-valine (176-V). This change results in increased binding of IgG1 and IgG3.[10] Fc γ RIIIB encodes a single GPI-linked polymorphic protein expressed on neutrophils. The allotypes designated as NA1 and NA2 are co-dominant and bi-allelic polymorphisms of the Fc γ RIIIB receptor that reflects a number of amino acid changes. Human SLE pedigree studies with genome-wide scans have mapped chromosome 1q21-24, in which the Fc γ RIIB is found to be an important locus. The genetic heterogenicity of the Fc γ receptor may have an impact on numerous autoimmune diseases and infections, especially lupus nephritis. Sequential Fc γ. receptor genetic association analysis of SLE patients disclosed as yet showed controversial or discrepant results across various study groups, suggesting that ethnic and geographical factors may cause various disease susceptibilities and complexities of SLE.

In both human and murine SLE, susceptibility allele has been mapped to intervals linked to the Fc γ RII gene on chromosome I and it has been hypothesized that the Fc γ RIIB promotor polymorphism may possibly predispose through germinal center B cells abnormally down regulating Fc γ RIIB1 expression upon autoantigen stimulation and thus escaping negative signals for IgG production. The differential activity of Fc γ RIIB alleles suggests a novel mechanism of Fc γ RIIB regulation that may influence the risk of autoimmune disease such as SLE. Results of genome wide linkage studies have suggested that the chromosomal region 1q23 is one of the strongest candidate regions for human SLE. Three Fc γ receptor II genes (Fc γ RIIA, Fc γ RII B, and Fc γ RII C) and two Fc γ RIII genes (Fc γ RIII A and Fc γ RIII B) have been physically mapped to a region of approximately 200 kb at 1q23 and are considered to be candidate susceptibility genes for SLE.[11]

Fc γ RII A polymorphism studies in Brazilian patients with immune complex-mediated SLE nephritis also showed over expression of the R131 allele with a significant increase in Fc γ RII A-R 131 homozygosity. The skewed distribution of Fc γ RII A with the predominance of homozygous R/R 131 genotype observed in Brazilian patients with lupus nephritis emphasize its importance as a heritable risk factor for immune complex-mediated renal injury in these patients.[6] Genotyping for Fc γ RIII B NA1/NA2 using allele specific primers in Korean patients showed an association of thrombocytopenic clinical involvement with Fc γ RIII B NA1/NA2 genotype and NA2 allele.[11] Thai patients with SLE also showed linkage disequilibria among Fc γ RII B, Fc γ RIII A, and Fc γ RIII B and suggested that the tendency of association of Fc γ RIII A could derive from linkage disequilibria with Fc γ RII B and Fc γ RIII B.[2]

A meta analysis report in Thai, Chinese, and Japanese populations in patients with SLE with nephritis and without nephritis suggests that the Fc γ RIIA-V/F 158 polymorphism has a significant impact on the development of lupus nephritis where a comparison of patients with lupus nephritis with patients with non nephritis SLE revealed a significant over presentation of the low binding F158 allele among patients with SLE who developed renal disease. F/F homozygotes had the highest risk of renal disease as compared with V/V homozygous.[2,3,12] A single nucleotide polymorphism in Fc γ RII B (695 T> C), coding for nonsynonymous substitution Ile232Thr (I232T), within the transmembrane domain in Japanese patients revealed that the frequency of the 232T/T genotype was significantly increased in patients with SLE.[12] The association of Fc γ RII B polymorphism with susceptibility to SLE in Chinese patients revealed a highly significant and independent association for Fc γ RII B and Fc γ RIII A genotypes where Fc γ RII A-H 131R and Ile 232 Thr (I232T) of Fc γ RII B and Fc γ RIII A-176V polymorphisms were found to be associated with lupus nephritis; these results suggested that Fc γ RII B is a common susceptibility factor to SLE nephritis.[13] Genotyping of Fc γ RII A 131 R/H, Fc γ RIII A- 176V/F, and Fc γ RIII B- NA1/2 polymorphisms may contribute to SLE disease susceptibility.[14]

Human Fc gamma receptors constitute a clustered gene family located on chromosome 1q23 that consists of Fc γ IIA, Fc γ IIB, Fc γ IIC, and Fc γ III B genes. Fc γ II B is unique in its ability to transmit inhibitory signals and recent studies have demonstrated a role for Fc γ II B deficiency in the development of autoimmunity as seen in cases of SLE. Genetic variations of Fc γ IIA, Fc γ IIA, and Fc γ IIIB and their association with clinical severity of the disease with SLE have been studied in Asian populations, but results are inconsistent. To examine the possibility that another susceptibility gene of primary significance exists within the Fc gamma receptor region and the association of these polymorphisms with clinical course of SLE needs to be elucidated from our large immunogenetically diverse population. Finally, a combination of defects in Fc γ IIA and IIIA were shown to be particularly deleterious, both in African American and Caucasian patients. However, the fact that only one defective variant is already associated with SLE indicates that these receptors have distinct functions in the development of autoimmunity.[7,8] Recent findings indicate that Fc γ RIIB is important in the initiation of autoimmunity by modulating activation of B cells and subsequent antibody production, thus contributing to the breakdown of immune tolerance. Fc γ RI and III seem to be crucial for disease expression and maintenance, probably by pro-inflammatory handling of autoantibody containing immune complexes.[16]

In an independent, large, case-controlled replication study, the association between Fc γ RIIIA and SLE was found to be stronger than the association of Fc γ RIIA with SLE. The data for Fc γ RIIIA satisfy the recommendations for evidence of linkage, a demonstrated association in both family-based and independent case-controlled cohorts, and functional relevance to disease as recently outlined for the definition of a true genetic effect.[17,18] It is proposed that there could be a common susceptibility gene in the Asian population. India has diverse populations, ethnic groups, and isolated tribal areas. In the Indian scenario, no studies have been undertaken to look for the association of Fc γ receptor polymorphism in patients with SLE and their role in pathophysiology of the disease. It will be interesting to know Fc γ receptor γ polymorphism in our country as no substantial data is yet available.

Activating and inhibitory Fc γ receptors seem to play an important role in the pathogenesis of SLE, both in initiation of autoimmunity and in subsequent development of inflammation. Though the mechanisms underlying the initiation of autoimmunity are yet to be elucidated, the ensuring development and maintenance of inflammatory processes could potentially be influenced at the level of Fc γ receptors. Modulating Fc γ receptor signaling mechanisms could influence the response to immune complexes and the course of the disease, making Fc γ receptors a potential candidate for immunotherapy. Characterization of Fc γ receptor genotypes, in conjunction with other properties of the humoral immune response such as antibody subclass and complement status, may provide essential insights into vaccine effectiveness and disease risk.

## References

[1] Baumann U, Schimidt RE, Gessner JE (2003). New insights into the pathophysiology and *in vivo* Function of IgG Fc receptors through gene deletion studies. Arch Immunol Ther Exp.

[2] Siriboonrit U, Tsuchiya N, Sirikong M, Kyogoku C, Bejrachandra S, Suthipinittharm P (2003). Association of Fc γ receptor IIB and IIIB polymorphisms with susceptibility of Systemic Lupus Erythematosus in Thais. Tissue Antigens.

[3] Chu ZT, Tsuchiya N, Kyogoku C, Ohashi J, Qian YP, Xu SB (2004). Association of Fc gamma receptor IIB polymorphism with susceptibility to systemic lupus erythematosus in Chinese: A common susceptibility gene in the Asian population. Tissue Antigens.

[4] Magnusson V, Johanneson B, Lima G, Odeberg J, Alarcon-Segovia D, Alarcon-Riquelme ME (2004). Both risk alleles for Fc gamma RIIA and Fc gamma R IIIA are susceptibility factors for SLE: A unifying hypothesis. Genes Immun.

[5] Hong CH, Lee JS, Lee HS, Bae SC, Yoo DH (2005). The association between Fc gamma RIIIB polymorphisms and systemic lupus erythematosus in Korea. Lupus.

[6] Bazilio AP, Viana VS, Toledo R, Woronik V, Bonfa E, Monteiro RC (2004). Fc gamma RIIA polymorphism: A susceptibility factor for immune complex-mediated lupus nephritis in Brazilian patients. Nephrol Dial Transplant.

[7] Reefman E, Limburg PC, Kallenberg CGM, Bijl M (2005). Fc γ receptors in the initiation and progression of systemic lupus erythematosus. Ann NY Acad Sci.

[8] Sullivan KE, Jawad AF, Piliero LM, Kin N, Luan X, Goldman D (2003). Analysis of polymorphisms affecting immune complex handling in systemic lupus erythematosus. Rheumatology.

[9] Bolland S, Yim YS, Tus K, Wakeland EK, Revetch JV (2002). Genetic modifiers of systemic lupus erythematosus in Fc gamma RIIB(-/-)mice. J Exp Med.

[10] Karassa FB, Trikalinos TA, Ioannidis JP (2003). The Fc gamma RIIIA- F158 allele is a risk factor for the development of lupus nephritis: A meta analysis. Kidney Int.

[11] Manger K, Repp R, Jansen M, Geisselbrecht M, Wassmuth R, Westerdaal NA (2002). Fc γ receptor IIA, IIIA and IIIB polymorphisms in patients with systemic lupus erythematosus: Association with clinical symptoms. Ann Rheumat Dis.

[12] Kyogoku C, Dijstelbloem HM, Tsuchiya N, Hatta Y, Koto H, Yamaguchi A (2002). Fc receptor gene polymorphisms in Japanese patients with systemic lupus erythematosus: Contribution of Fc gamma RIIB genetic susceptibility. Arthritis Rheum.

[13] Pan F, Ye D, Zhang K, Li X, Xu J, Chen H (2007). The combination of ILE225THR polymorphism of Fc γ receptor IIB gene and hypersensitiveness as risk factor for human systemic lupus erythematosus in Chinese populations. Indian J Dermatol.

[14] Hatta Y, Tsuchiya N, Ohashi J, Matsushita M, Fujiwara K, Hagiwara K (1999). Association of Fc γ receptor IIIB but not of Fc γ receptor IIA and III A, polymorphism with systemic lupus erythematosus in Japanese. Genes Immun.

[15] Tsuchiya N, Kyogoku C (2005). Role of Fc γ receptor IIB polymorphism in the genetic background of systemic lupus erythematosus. Autoimmunity.

[16] Su K, Wu J, Edberg JC, Li X, Ferguson P, Cooper GS (2004). A promoter haplotype of the immuno receptor tyrosine based inhibitory motif bearing Fc γ receptor IIB alters receptor expression and associates with autoimmunity, I: Regulatory Fc γ receptor IIB polymorhphisms and their association with systemic lupus erythematosus. J Immunol.

[17] Sardjono CT, Mottram RL, Hogarth PM (2003). The role of Fc γ IIA as an inflammatory mediator in rheumatoid arthritis and systemic lupus erythematosus. Immunol Cell Biol.

[18] Dahlman I, Eaves IA, Kosoy R, Morrison VA, Heward J, Gough SC (2002). Parameters for reliable results in genetic association studies in common disease. Nat Genet.

